# Host Call Frequency Drives Attractiveness to an Eavesdropping Micropredator

**DOI:** 10.1002/ece3.74381

**Published:** 2026-09-20

**Authors:** Timothy P. Cutajar, Alistair G. B. Poore, Jodi J. L. Rowley

**Affiliations:** ^1^ Centre for Ecosystem Science, School of Biological, Earth and Environmental Sciences UNSW Sydney Sydney New South Wales Australia; ^2^ Australian Museum Research Institute, Australian Museum Sydney New South Wales Australia

**Keywords:** acoustic preferences, eavesdrop, frequency, frog‐biting midge, sensory ecology, signal evolution

## Abstract

Acoustic sexual signals are vulnerable to interception by eavesdropping predators, micropredators and parasitoids. Signal traits can impact attractiveness to eavesdroppers and potential mates alike. Thus, signals are influenced by interacting selection pressures and drive predator/prey interactions and their consequences (e.g., disease dynamics where eavesdroppers are vectors). We assessed the spectral preferences of eavesdropping micropredators in a near cosmopolitan system—frog‐biting midges (*Corethrella* spp.) and frogs—for the first time in Oceania. Frequency treatments that spanned the spectral profile of the study area's frog fauna (sinusoidal pure tones at 0.1, 0.3, 0.5, 0.8, 1.4, 2.3 and 3.2 kHz) were offered in situ in no‐choice experiments. Frequencies of 0.5–1.4 kHz attracted significantly more midges than silent controls with a peak at 0.8 kHz (74 mean flies vs. 0.36 to control). The lower spectral limit of attractiveness to *Corethrella* closely matched the lowest‐calling local frog species, and was higher than in other regions where even lower‐calling frogs occur, suggesting potential evolutionary adjustment of *Corethrella*'s preferred spectral profiles to what is available. The smallest‐bodied frogs called above the frequency range that attracted *Corethrella*, and we posit that this may avoid negative selection pressures that are conceivably stronger for smaller hosts, rather than being purely a result of small bodies' biomechanical constraints. Our results are consistent with broader observations that eavesdroppers and intended receivers commonly share preferences, and competing selection pressures are likely incurred by intended and unintended signal receivers, with implications for signal evolution, disease dynamics and potentially biodiversity conservation.

## Graphical abstract

Acoustic sexual signals are vulnerable to interception by eavesdropping unintended receivers, and signals are thus influenced by interacting selection pressures. Frequencies of 0.5–1.4 kHz attracted frog‐biting midges (*Corethrella* spp.) and closely matched the lowest‐calling local frog species. Our results suggest potential evolutionary adjustment of *Corethrella*'s preferred spectral profiles to what is available, and that very small frogs may evolutionarily adjust calls to avoid attracting *Corethrella*.

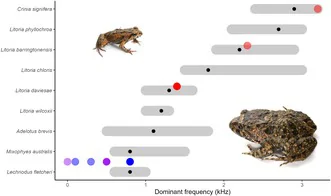

## 1 Introduction

Eavesdropping, when intraspecific signals are detected and exploited by non‐target receivers, is a common tactic among predators (McGregor 1993; Zuk and Kolluru 1998). Visual, chemical, or acoustic signals such as those conveying territoriality, fitness or reproductive readiness are vulnerable to interception (Daly 1978; Endler 1980; Meuche et al. 2016). Non‐target receivers are particularly prevalent in acoustic signalling systems (Meuche et al. 2016; Zuk and Kolluru 1998). For example, vertebrate predators including some bat (Belwood and Morris 1987; Tuttle and Ryan 1981), lizard (Sakaluk and Belwood 1984) and bird species (Bell 1979; Zuk and Kolluru 1998), along with many micropredator flies (Allen 1995; Bernal et al. 2006; Soper et al. 1976), are phonotactic (attracted by sound) and eavesdrop on the calls of their prey or hosts.

The taxonomic diversity of acoustic signal eavesdroppers is mirrored by the signallers themselves, which include invertebrates and both aquatic and terrestrial vertebrates and are at greater risk of interception than other signaller types (White et al. 2022). Thus, non‐target receivers have high potential to impact the evolution of prey and host signals across acoustic signalling systems. Eavesdropping predators directly prey upon organisms sending acoustic signals (Tuttle and Ryan 1981) or reduce the fitness of potential prey through signal interruption and costly predator avoidance (Tuttle et al. 1982). Similarly, eavesdropping micropredators that feed on host tissues impact fitness through blood loss and the transmission of toxins and diseases (Oxer and Ricardo 1942; Pfäffle et al. 2009; Thompson et al. 2010), and when hosts' time and energy are diverted to avoidance (Leavell et al. 2022) or grooming behaviours (Hawlena et al. 2007; Mooring 1995).

Identifying what traits of acoustic signals are attractive to phonotactic eavesdroppers, and therefore shaped by the selection pressures they incur, helps understand sexual signal evolution. The preferences of both eavesdroppers and target receivers (i.e., potential mates) are often shared and can be affected by call frequency (Amorim et al. 2015; Gerhardt 2005; Shaw and Herlihy 2000), temporal features (Amorim et al. 2015; Marcel et al. 1991; Shaw and Herlihy 2000; Tárano and Fuenmayor 2013) and complexity (Marcel et al. 1991; Michael J. Ryan 1985). The selection pressure on attractive calls is thus an interaction between potentially opposing pressures from target and non‐target receivers. Understanding eavesdropper preferences could therefore inform specific and competing drivers of host and prey signal characteristics, especially when considered in the context of mate choice (Virgo et al. 2019).

Eavesdroppers that respond to signals from frogs are useful systems for investigating acoustic preferences. With just a handful of exceptions, nearly all sexual signals by frog species are acoustic, with call variation both among (Oldham and Gerhardt 1975) and within species (Bee 2002; Signorelli et al. 2014) potentially subject to target and non‐target receivers' preferences. An array of phonotactic eavesdroppers has evolved to prey on frogs and use male advertisement calls in the process (White et al. 2022). The most abundant and ubiquitous eavesdroppers of frogs' acoustic signals are frog‐biting flies; diverse and widespread lineages of corethrellid, culicid and sycoracine flies whose females locate frogs acoustically to take blood meals (Borkent and Belton 2006; Borkent 2008; Cutajar and Rowley 2020). Further, frog‐biting flies are known to exhibit preferences during host selection across multiple acoustic variables (Bernal et al. 2006; Aihara et al. 2016; Meuche et al. 2016; Virgo et al. 2019; Legett et al. 2021).

The preferences of *Corethrella* and *Uranotaenia* have been investigated in several studies across a range of acoustic variables and regions. In the few sites frequency preferences have been investigated, frequencies at the lower end of available ranges were most attractive to both genera, with maximum attractive frequencies ranging from 0.8 to 3 kHz (Legett et al. 2021; Meuche et al. 2016; Virgo et al. 2019). There may be general preferences for complex calls, but this has been investigated somewhat inconsistently among studies, using the presence versus absence of harmonics (Legett et al. 2021), or the addition of a second call type to typical calls (Aihara et al. 2016; Bernal et al. 2006). High call repetition rates seem to be preferred by *Corethrella* (Aihara et al. 2016; Meuche et al. 2016). Their preferred call durations may be within 125–500 ms (Meuche et al. 2016; Virgo et al. 2019), but differences in how this is measured among regions preclude broad pattern identification.

Frog‐biting flies likely impact frogs' fitness, evolution and conservation through multiple deleterious, yet poorly understood pathways. Camp (2006) theorised that, though individually small, multiple flies' blood meals can conceivably total almost one third of a hosts' blood. Even where blood loss may be minimal, frogs irritated by fly bites pause calling and exhibit defensive behaviours (Virgo et al. 2019). Since female frogs tend to prefer calls with a higher proportion of sound relative to silence during calling (e.g., Gerhardt et al. 2000), any call interruption is likely to affect reproductive fitness.

In addition to blood loss and calling interruption, frog‐biting flies likely mediate frogs' acoustic signal evolution via disease, to which frogs are highly vulnerable worldwide (Luedtke et al. 2023). *Corethrella* transmit trypanosome blood parasites among frogs (Borkent 2008), as likely do other frog‐biting flies (Bernal and Pinto 2016). Trypansomes' pathogenic effect on wildlife is virtually unstudied (but see Bardsley and Harmsen 1973; Megía‐Palma et al. 2024) but trypanosomes are highly diverse and can be extremely pathogenic, causing mortality in humans (reviewed in Vogel and Schaub 2021). *Corethrella* can carry DNA of the amphibian chytrid fungus (*Batrachochytrium dendrobatidis* (*Bd*)), and may act as vectors for *Bd*‐caused chytridiomycosis, the greatest wildlife pandemic disease known (Toledo et al. 2021). Therefore, as a major driver of frog‐biting flies' host selection, call frequency of frogs likely has considerable, albeit unknown, impacts on amphibians' disease dynamics and potentially their conservation status.

Frog‐biting flies are emerging as a model for understanding eavesdropper ecology, but current knowledge on their acoustic preferences is too sparse, taxonomically, geographically and methodologically, to confirm many patterns among lineages or regions. For example, such information—even on basic spectral preferences—is completely lacking for Oceania, despite it being the only region from which *Corethrella*, *Uranotaenia* and phonotactic *Sycorax* are all known (Borkent 2008; Cutajar and Rowley 2020; Wilkerson et al. 2021). Although *Uranotaenia* occurs in Australia (Wilkerson et al. 2021), no Australian species is known to be phonotactic. Both *Corethrella* and *Sycorax* were only recently confirmed to be phonotactic in Australia (Cutajar and Rowley 2020), but acoustic preferences are unknown. Addressing this regional gap enables us to exploit frog‐biting flies' near cosmopolitan distribution, positioning them as a global model for understanding eavesdropper ecology and its impacts on signaller evolution.

We aimed to examine the spectral preferences of three frog‐biting fly lineages (*Corethrella*, *Uranotaenia* and *Sycorax*) in Australia. Knowledge of these lineages' acoustic preferences will contribute to our understanding of sexual signal evolution by assessing the potential evolutionary outcomes of interacting selection pressures incurred by target and non‐target receivers in the contexts of mate choice and disease.

## 2 Methods

### 2.1 Study Sites and Period

**FIGURE 1:**
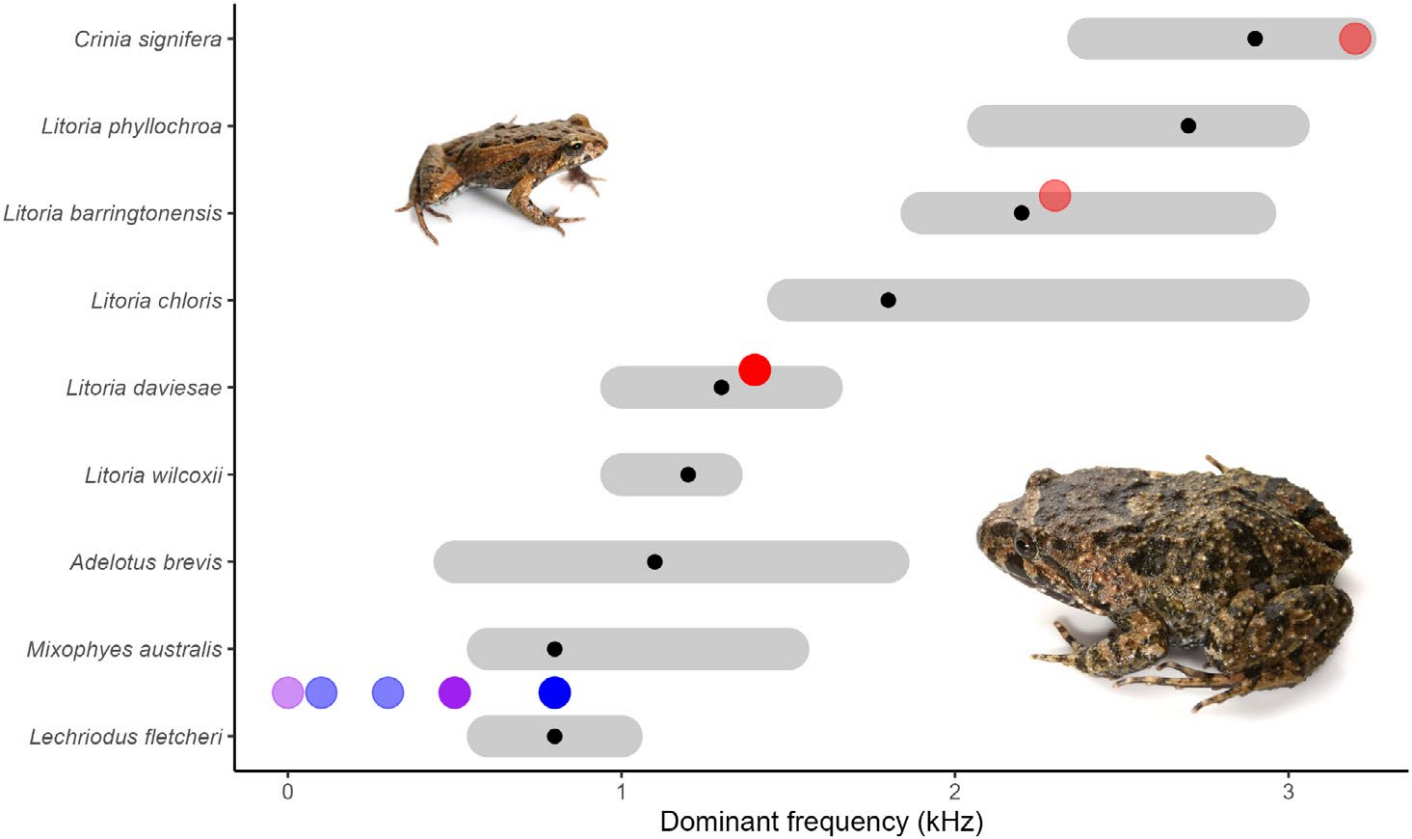
Mean dominant frequencies (black dots) and mean frequency ranges (grey bars) of frog species known from the study area in Barrington Tops National Park and Chichester State Forest, NSW Australia. Coloured dots represent experimental frequency treatments during the first experimental phase (red), both phases (purple) and second phase (blue). Opaque treatments attracted significantly more frog‐biting flies than the silent control, and semitransparent treatments did not. The highest‐ and lowest‐calling frog species are pictured to scale at the top left ( *Crinia signifera* ) and bottom right ( *Adelotus brevis* ).

We investigated which sound frequencies attract the most frog‐biting flies at four sites in Barrington Tops National Park and Chichester State Forest in eastern New South Wales, Australia, during the late austral spring to early autumn from November 2022 to March 2023. The study area is hilly and characterised by a mix of mostly wet sclerophyll and rainforest. Sites 1 (−32.16, 151.49; 480 masl), 2 (−32.14, 151.48; 400 masl) and 3 (−32.13, 151.47; 500 masl) in this study corresponded to sites 3, 2 and 5 in Cutajar and Rowley (2020) (see map in Figure 1 of that study), respectively, where *Corethrella* and *Sycorax* are abundant, show phonotaxis to frog calls and were commonly observed attacking frogs. Site 4 in this study was a nearby tributary of the Allyn River (−32.13, 151.48; 470 masl). All sites were at streams in rainforest and were 0.5–4 km apart. A small study area helped mitigate potential habitat variation, which can impact species composition and host associations in frog‐biting flies (Grafe et al. 2019). Sampling was limited to three times per 10 day period at each site. This was done as numerous consecutive trapping sessions at a single site can reduce trap yield (Meuche et al. 2016). We performed an average of 14.4 acoustic tests every month during this period for a total of 72, distributed equally across sites and treatments.

### 2.2 Frequency Tests

We performed no‐choice experiments following Ambrozio‐Assis et al. (2019) to determine the relative attractiveness of frequency treatments to frog‐biting flies (measured as the number of flies trapped). For all experiments, we used an E702 (Encephalitis Vector Survey) fan‐powered suction trap (Australian Entomological Supplies, South Murwillumbah, Australia) with a speaker (W‐King D8 Wireless Speaker; W‐King, Shenzhen, China) broadcasting an acoustic attractant (McKeever and Hartberg 1980). As attractants, we used sinusoidal pure tones synthesised using Audacity 3.0.5 software (https://www.audacityteam.org/) instead of recorded frog calls. *Corethrella* are attracted to pure tones, provided there are silent intervals (Virgo et al. 2019; Meuche et al. 2016), enabling us to examine the effect of frequency without unintended variation in other call features (e.g., call rate or complexity) confounding results.

We ran the no‐choice experiments as a randomised block design, with one of each frequency treatment run at each site, but each running on different nights with the order randomised. Traps were set so that their openings were approximately 50 cm above the ground, as previous tests showed the highest sound attractiveness when broadcasted near the ground (0.4 m; Meuche et al. 2016). Frequency treatments included a no sound control (speaker broadcasting a blank sound file) and seven treatments. Identical except in their frequency, all treatments consisted of a 30 s wav file in which alternate 250 millisecond (ms) pure tones and 250 ms inter‐pure tone intervals (both within the most attractive ranges for *Corethrella* in Costa Rica (Virgo et al. 2019)) played on repeat. All treatments were broadcast at a standard volume of 80 dB SPL (re. 20 μPa) measured at 1 m with a SPL meter (SKU IC‐CENTER32; CENTER Technology Corp, Taipei, Taiwan), following most studies of frog‐biting flies' acoustic preferences (e.g., Legett et al. 2021; Virgo et al. 2019). We ran treatments for 2 h each between sunset and 02:00 h, alternating sampling start time between treatments to account for any effect of time on fly activity.

It is possible that local frogs called during experiments, and such calls can reduce the number of flies caught using acoustic attractants (Borkent 2008). All frog species known in the study area (defined in Cutajar and Rowley 2020) call throughout the study period (Rowley and Callaghan 2020). Any effects of nearby calling cannot confound the experiment with frequency treatments randomly allocated to trials and differences in calling at a particular site and time accounted for via the random factors included in statistical models (see Section 2.3).

The spectral preferences of frog‐biting flies in Australia were entirely unknown, so we divided the seven frequency treatments across two testing phases, with results from the first phase informing the frequencies we used as treatments in the second. To determine treatments used during phase one we analysed the calls of local frog species and selected four treatments that span the resulting community‐level spectral profile of calls: 0.5, 1.4, 2.3 and 3.2 kHz. For methodological details of that analysis see Figure 1 and Appendix A.

To increase resolution and determine a lower frequency limit in attractiveness, treatments used during the second testing phase were 0.1, 0.3, 0.5 and 0.8 kHz. We continued to use 0.5 kHz during phase two as it consistently attracted frog‐biting flies throughout phase one and thus served as a standard positive control, which could be used to determine whether differences in trapping success between the two phases were explained by frequency or some other variable, for example fly seasonality. We selected 0.1 and 0.3 kHz because during phase one, flies showed a preference for 0.5 kHz—the lowest‐frequency treatment. We also selected 0.8 kHz to determine whether phase one tests had missed a possible peak attractiveness at some frequency between 0.5 and 1.4 kHz, which also successfully collected flies, albeit fewer than 0.5 kHz.

We collected flies from traps after each trapping session, transported them by car for 20–30 min and transferred them to a freezer at −20°C for at least 4 h for euthanasia. We differentiated fly morphospecies by gross morphology and pigmentation and counted the number of individuals of each morphospecies trapped by each acoustic treatment. We did not identify flies to species level because the taxonomy of frog‐biting flies in Australia is poorly known and we likely collected undescribed species requiring further taxonomic treatment. We pooled specimens by morphospecies and trapping session (2 h running of a single trap and treatment) for storage. Specimens were stored in molecular‐grade ethanol for future molecular analysis. After fieldwork, we transported specimens to the Australian Museum, where they will be deposited.

We ran 96 trapping sessions (2 h single trap runs) in total. After the removal of 24 sessions from before November 2022 (which yielded insufficient flies: see results), 72 remained in the dataset. Of these, there were seven sessions each at 0.1, 0.3, 0.8, 1.4, 2.3 and 3.2 kHz, 16 at 0.5 kHz and 14 using the silent control.

### 2.3 Statistical Analysis

The responses of frog‐biting fly morphospecies to our frequency treatments were analysed separately with generalised linear mixed models, with the number of individuals collected as the dependent variable, frequency treatment as a fixed factor and site and month as random factors, using a negative binomial distribution in the *glmmTMB* package in R (Brooks et al. 2025). We did not analyse morphospecies collected during < 10% of trapping events or if fewer than 30 individuals were collected in total due to their small sample size. Model diagnostics were checked with the R package *DHARMa* (Hartig 2022) and statistical inference derived from analyses of deviance contrasting models with and without the fixed factor. *Post hoc* tests contrasting all frequencies and the control were run in the R package *emmeans* (Lenth et al. 2024). We pooled response data from treatment phases one and two for analysis to enable direct comparisons among all frequency treatments. Longer‐term variation in weather conditions among our sampling periods was accounted for by the random factor month, but we further explored the potential for temperature and humidity to explain observed results by performing separate analyses of deviance that contrasted models with temperature or humidity as fixed factors and site as a random factor, and the model with only site as the random factor.

### 2.4 Ethical Note

This research was carried out in accordance with permits to access biological resources issued by the NSW Government under the Biodiversity Conservation Act 2016 (SL102144) and permission from the Australian Museum Animal Ethics Committee (21‐03). Sinusoidal pure tone attractants, in addition to being more useful for our study than recorded frog mating calls, which are typically used for attracting frog‐biting flies, also had the benefit of being less likely recognisable by frogs. Thus, any potential impact on local frogs' reproductive activities was likely reduced. To reduce disturbance to both fly and frog populations we limited trapping to three times per 10‐day period at each site. To reduce disturbance to collected flies, we selected accommodation as close as possible to field sites, thus reducing the time they were held alive in captivity.

## 3 Results

### 3.1 Frequency Tests

**FIGURE 2:**
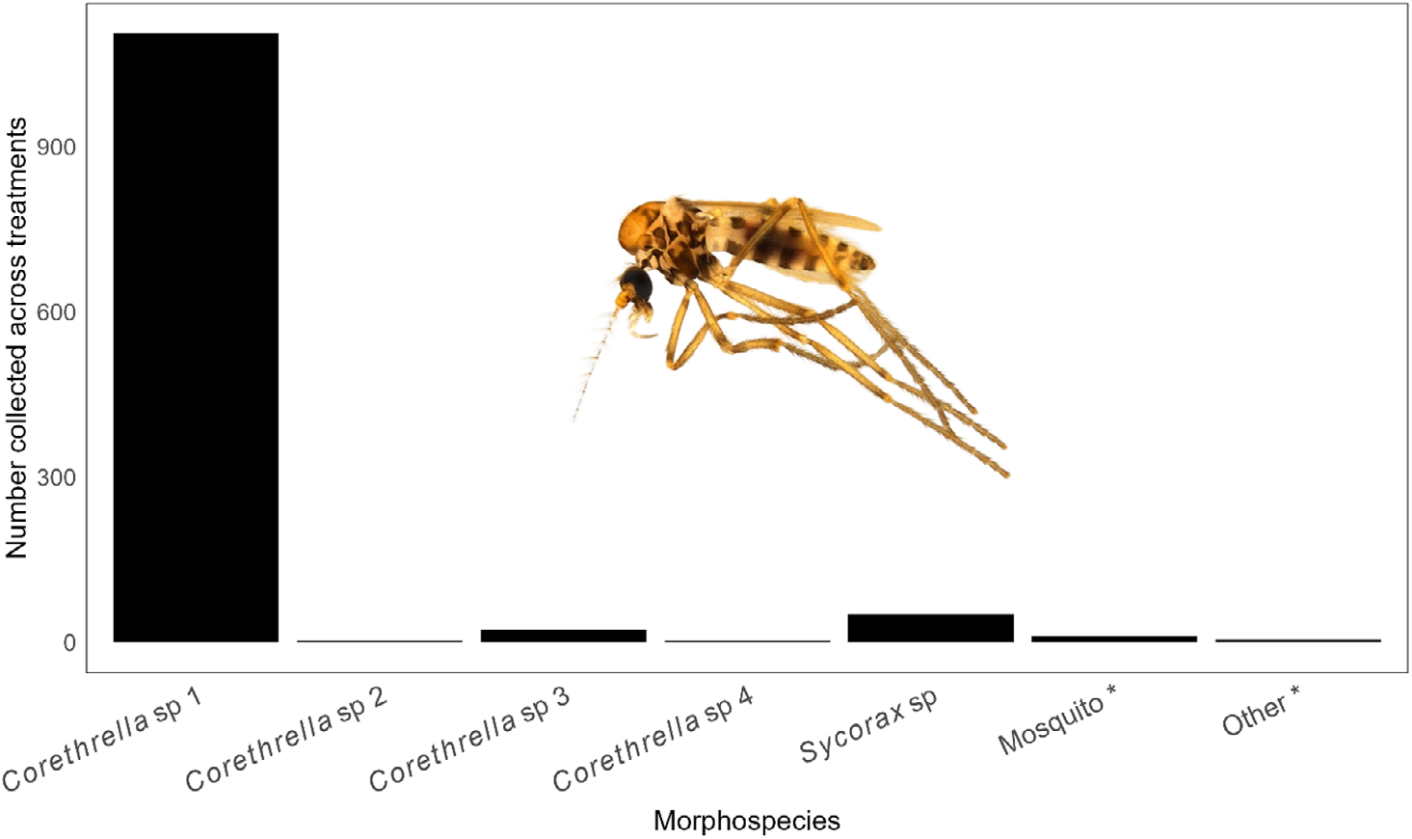
Total number of individuals of each biting fly group collected across all frequency treatments in Barrington Tops National Park and Chichester State Forest and insert image of *Corethrella* sp. 1. Groups not known to be phonotactic in Australia are denoted by an asterisk.

A total of 1201 biting flies was collected from November 2022 to March 2023, including 1186 of four morphospecies of known frog‐biting genera (four *Corethrella* and one *Sycorax*), nine mosquitos not known to be phonotactic in Australia and five other, unidentified biting flies (Figure 2). No *Uranotaenia* were collected. Flies within the ‘mosquito’ and ‘other’ groups were kept pooled, not identified to genus level, but none were collected in sufficient numbers for analysis.

**FIGURE 3:**
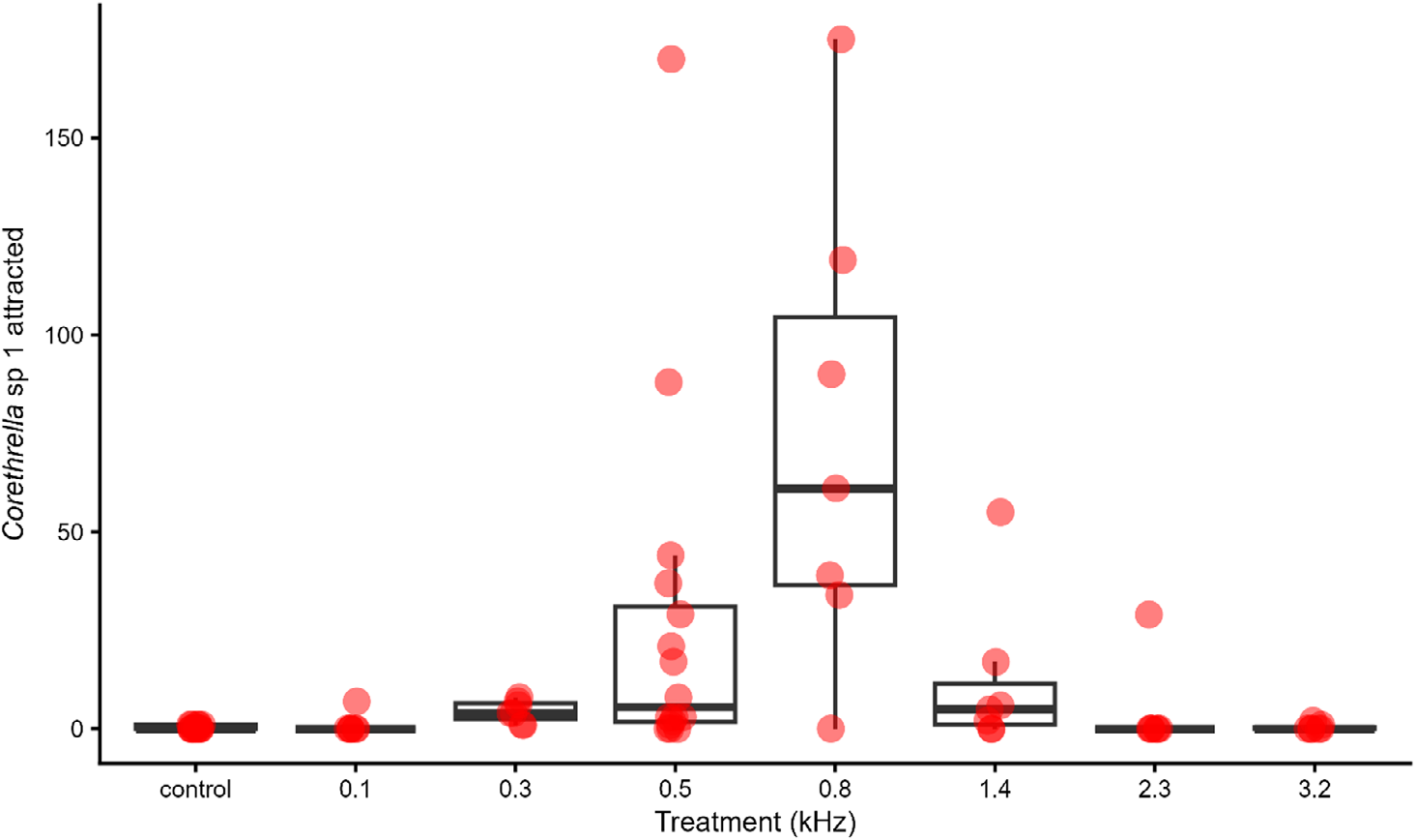
The number of individuals of a frog‐biting midge (*Corethrella* sp. 1) in treatments that differed in frequency (in kHz) of sinusoidal pure tones in Barrington Tops National Park and Chichester State Forest.

**FIGURE A1:**
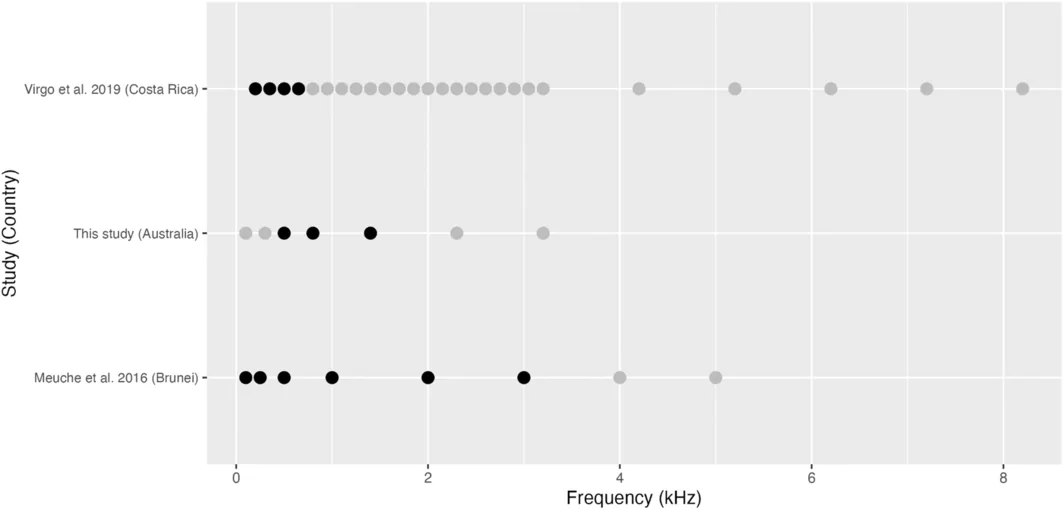
Frequencies that have been used as treatments in tests of spectral preference in *Corethrella*. Black dots represent frequencies that were significantly preferred and grey dots represent those that were not, but that may have still attracted midges in smaller numbers. Only studies that used sinusoidal pure tones are included, thus avoiding potentially confounding variables in natural frog calls.

**FIGURE A2:**
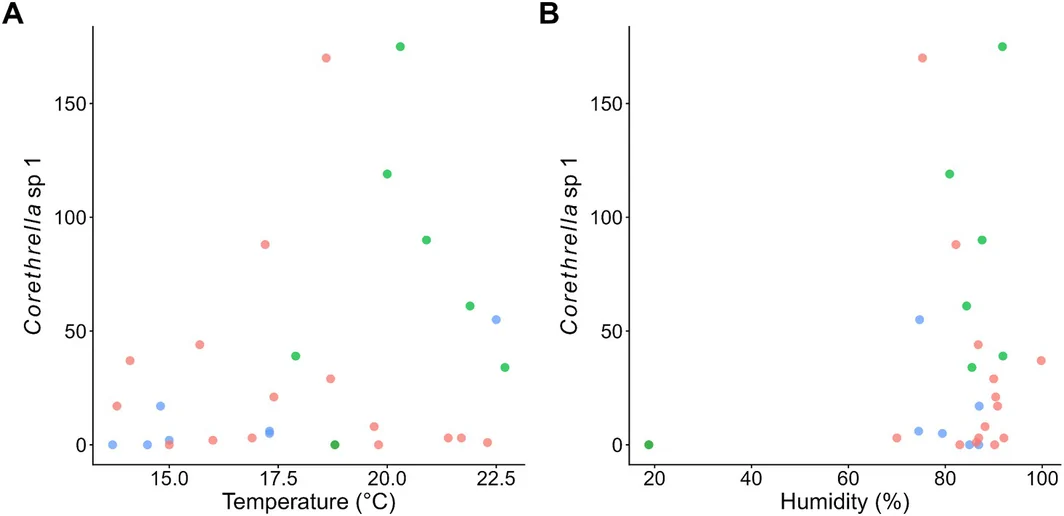
The number of *Corethrella* sp. 1 trapped in contrast to temperature and humidity on sampling nights using treatments that attracted significantly more than the silent control: 0.5 kHz (red), 0.8 kHz (green) and 1.4 kHz (blue).

Only *Corethrella* sp. 1 was collected in large enough numbers for statistical analyses (Figure 2). Analysis of all treatment responses (phases one and two pooled) revealed a significant effect of frequency on the number of *Corethrella* sp. 1 collected during trapping events (Figure 3, χ^2^ = 45.22, df = 7, *p* < 0.001). Three frequency treatments attracted significantly more midges than the silent control (Figure 3): the model‐estimated capture rate was 66‐fold higher at 0.5 kHz with mean (and max) of 26.625 (170) (*n* = 16, CI = 7.55–68.76, *p* < 0.001), 292‐fold higher at 0.8 kHz with mean (and max) of 74 (175) (*n* = 7, CI = 15.32–655.22, *p* < 0.001) and 28‐fold higher at 1.4 kHz with mean (and max) 12.143 (55) (*n* = 7, CI = 1.64–55.87, *p* = 0.001), with no evidence that the other frequency treatments differed from the control: 0.1 kHz 3‐fold higher than control (*n* = 7, CI = 0.22–5.27, *p* = 0.95), 0.3 kHz 18‐fold higher (*n* = 7, CI = 1.06–35.94, *p* = 0.08), 2.3 kHz 6.29‐fold higher (*n* = 7, CI = 0.19–25.01, *p* = 0.21) and 3.2 kHz 0.9‐fold higher (*n* = 7, CI = 0.04–2.46, *p* = 1) (Figures 3 and A1). Although the 0.8 kHz treatment attracted the highest mean number of flies, pairwise contrasts did not find evidence that this frequency differed significantly from the 0.5 and 1.4 kHz treatments (*p* = 0.93 and 0.8, respectively). There was no evidence for differences among frequency treatments for those treatments that did not differ significantly from the control (0.1, 0.3, 2.3 and 3.2 kHz; all pairwise comparisons *p* ≥ 0.56) (Figure 3). There was no evidence for an effect of either temperature (β = 2.00, 95% CI = −0.84 to 4.84; χ^2^ = 1.88, df = 1, *p* = 0.171) or humidity (β = 0.17, 95% CI = −0.49 to 0.84; χ^2^ = 0.27, df = 1, *p* = 0.604) on the numbers of *Corethrella* collected (Figure A2).

## 4 Discussion

We provide the first analysis of acoustic prey localisation of any frog‐biting fly lineage outside Asia and the Americas. We determined the spectral preferences of an Australian frog‐biting midge (*Corethrella*) species and identified a frequency that attracted that species in large numbers. Our results contribute to the scant but growing literature on the sensory ecology of frog‐biting flies globally.

Frequencies at 0.5–1.4 kHz were attractive to *Corethrella*, with a peak at 0.8 kHz, whereas frequencies above and below this range did not attract significantly more than the control. Although only 0.5, 0.8 and 1.4 kHz differed significantly from the control, uncertainty varied among treatments. The 0.3 kHz treatment showed a moderate estimated increase relative to the control despite not differing significantly; additional sampling may have clarified whether weak attraction occurs at that frequency. Similarly, whereas humidity showed a negligible effect size with relatively narrow confidence intervals, suggesting genuinely absent effects, temperature estimates were less certain and moderate effects could not be excluded with the current sample size.

In contrast to this study, sounds that attract *Corethrella* elsewhere include lower frequencies than these: 0.2–0.8 kHz out of up to 8.2 kHz in Costa Rica (Virgo et al. 2019) and 0.1–3 kHz out of up to 5 kHz in Brunei (Meuche et al. 2016). In Japan, 1.8 kHz was preferred over 3 kHz, though lower frequencies than 1.8 kHz were not tested and frequency interacted with other variables (Legett et al. 2021). In all these studies except ours, the lowest frequency option was attractive. Our multi‐phase sampling enabled treatment adjustment to determine not only the upper frequency attractiveness limit, but also the lower.

Interestingly, the lowest frequency that attracted *Corethrella* (0.5 kHz) matched the lowest dominant frequency found for any of the nine local frog species analysed (*Adelotus brevis*; Figure 1). *A. brevis* is a frequent host of *Corethrella* (Cutajar et al. in prep). This may indicate the limit to flies' preferences for low frequencies mirroring the available host species alongside which they evolved and warrants further investigation. Although its minimum dominant frequency is ~0.5 kHz, *A. brevis* ' call is broadband, often reaching spectral lows ≤ 0.3 kHz (Cutajar et al. in prep), a larger sample size of which treatment in this study might have revealed a minor effect of attraction in *Corethrella*. This is supported by the presence of frogs with lower‐frequency calls than that of *A. brevis* in frog and *Corethrella* assemblages elsewhere, in which even lower calls were preferred. Examples are *Leptodactylus savagei* in Costa Rica (Virgo et al. 2019), with dominant frequencies as low as 0.35 kHz and fundamental frequencies even lower (Heyer 2005) and *Polypedates colleti* in Brunei, with a mean dominant frequency of 0.67 kHz, but individuals commonly calling at far lower frequencies (Meuche et al. 2016).

Our results highlight the possibility that call frequency may play a role in the disease ecology of amphibians. Frequencies attractive to *Corethrella* matched local frog species whose calls were low to medium‐pitched. Given that body size and call frequency are highly negatively correlated in frogs (Duellman and Trueb 1994; Gerhardt and Huber 2002; Wells 2013), this indicates that small‐bodied frogs in this region may face a lower risk of disease transmission by frog‐biting flies. Indeed, calls of two of the study area's smallest species, *Litoria phyllochroa* and *Crinia signifera* , are well above the range of frequencies that attracted *Corethrella* (starting at 2.3 kHz, Figure 1). This is a pattern seen elsewhere; glass frogs (Centrolenidae) are among the smallest neotropical frogs and typically call at 4–7 kHz (Brunner 2022)—far above what attracted *Corethrella* in Costa Rica (Virgo et al. 2019). In Borneo too, one of the world's smallest frogs ( *Microhyla nepenthicola* ) calls at 3.0–5.5 kHz (Das and Haas 2010): just above the range that attracted *Corethrella* in that region (Meuche et al. 2016).

It would be interesting to determine whether some small frogs evolutionarily exaggerate high‐pitched calls in response to eavesdroppers' preferences for low to medium frequencies. Biomechanical constraints related to body size play an important role in call frequency (Gerhardt and Huber 2002; Martin 1972; Nevo and Schneider 1976), but the relationship is not equally linear among species and is impacted by other factors relating to signal perceptibility, masking and competition (Amézquita et al. 2006; Brumm and Slabbekoorn 2005; Hoskin et al. 2009). *Corethrella* transmit trypanosome blood parasites and frogs' call attractiveness to them is positively correlated with infection load (Meuche et al. 2016). The pathogenesis of trypanosomes in frogs is poorly studied but can be lethal (Bardsley and Harmsen 1973). *Corethrella* may also transmit the pandemic‐causing amphibian chytrid fungus (Toledo et al. 2021) and even uninfected *Corethrella* incur a cost to their host in the form of blood loss (Camp 2006). A given volume of lost blood or infection load size conceivably represents a greater risk for smaller‐bodied frogs, which may be under greater selective pressure to evolve calls outside the attractive range for *Corethrella*. Indeed, in the diminutive Bornean frog *Metaphrynella sundana* , often attacked by *Corethrella* (Grafe et al. 2008, 2019), intraspecific variation in dominant frequency is high and high‐frequency calls are unexpectedly common despite being unattractive to females (Lardner and Lakim 2004).

Results from this and similar studies (Meuche et al. 2016; Virgo et al. 2019; Legett et al. 2021) demonstrate that, like female frogs, *Corethrella* tend to prefer calls at the lower end of what is available from local male frogs (Ryan and Keddy‐Hector 1992; Mclean et al. 2012). In turn, such shared preferences between intended and unintended receivers are common in many acoustic signalling systems including insects, fish and birds (Amorim et al. 2015; Gerhardt 2005; Shaw and Herlihy 2000). Of the four frog species confirmed by diet analysis to be parasitised by *Corethrella* in our study area (Cutajar and Rowley 2020), females' acoustic preferences have been tested only for *Litoria wilcoxii* . In that species, low frequency calls are moderately preferred during two‐choice tests where options represent variation found in nature (Schwenke 2022). Though a single species, *L. wilcoxii* is a good representative for investigating the potentially interacting preferences of its calls' target and non‐target receivers. Its females' preference for lower calls is consistent with trends among frogs generally (Ryan and Keddy‐Hector 1992; Mclean et al. 2012), and it was by far the most preyed‐on species by *Corethrella* in our study area (Cutajar and Rowley 2020) as well as in a much more species‐rich frog community in southeast Queensland (Cutajar et al. in prep). This is further supported anecdotally by frequent observations of *Corethrella* preying on calling male *L. wilcoxii* (TC pers. obs.).

A moderate, rather than absolute preference among female *L. wilcoxii* for lower calls is interesting. Though Schwenke (2022) did not consider eavesdroppers, it seems possible that in this highly preyed‐on species, varying female mate choice validates either of two reproductive strategies. In a ‘live fast, die young’ strategy, males with lower calls attracting more females may have higher reproductive output per season, but over fewer seasons because they also attract more flies. Conversely, a ‘live slow, die old’ strategy might see males call at higher frequencies resulting in lower seasonal output but over more seasons. If this is the case, the spread of the amphibian chytrid fungus (regardless of flies) has likely impacted the relative frequency of such strategies. Chytridiomycosis reduces average lifespan in infected populations, so its arrival would likely favour those individuals already adopting a ‘live fast, die young’ approach—that is unless frog‐biting flies play an active role in its spread. However, better understandings of frog‐trypanosome infection dynamics and whether frog‐biting flies represent a significant infection pathway for *Bd* would be needed to infer which, if any, of these effects are likely.

Although we attracted large numbers of *Corethrella* during this study, other frog‐biting fly groups were not well sampled. It is unclear why so few *Sycorax* were collected in our traps. This group bites frogs globally (Bravo et al. 2010; Cutajar and Rowley 2020; Mačát et al. 2015) and, uniquely to Australia, is known to be phonotactic there. Indeed, this was demonstrated at three of our specific study sites, where Cutajar and Rowley (2020) attracted large numbers with call recordings of local species *Mixophyes australis* and *Litoria barringtonensis* (Cutajar and Rowley 2020). Anecdotally, we also observed them in large numbers during fieldwork for this study and others. It appears that something is missing from the sinusoidal pure tones used in this study that Australian *Sycorax* use to home in on natural frog calls—perhaps spectral and/or temporal complexity, amplitude modulation, or simply a frequency outside the range we tested (e.g., non‐dominant frequencies at the upper or lower extremes of calls' ranges, which *Sycorax* may be aurally filtering from true frog calls).

*Uranotaenia* are poorly known in Australia. Records of the genus are sparse—only 13 records for Australia in the national biodiversity data aggregate (ALA 2024). Those records are from both north and south of our study area, and the genus's true absence would be surprising, although we did not collect any during this study. Virgo et al. (2019) and Meuche et al. (2016), who also used pure tones, did not report the collection of *Uranotaenia* either, though the latter did, like us, collect small numbers of mosquitos that did not show any evidence of having been attracted by the tones. Perhaps *Uranotaenia* share with the Australian *Sycorax* the need for some yet unknown auditory cue missing in sinusoidal pure tones. However, Cutajar and Rowley (2020), who used natural recorded frog calls at the same sites, also did not collect *Uranotaenia*.

Further research is needed to comprehensively characterise frog‐biting flies' acoustic preferences globally to better understand eavesdroppers' sensory ecology, the evolution of sexual signals and possibly even disease ecology. Preferred frequencies in other areas co‐analysed with local frog fauna's call characteristics would shed light on the potential strength of links between the evolution of preference for low frequencies and the specific stimuli available to them. Direct co‐analyses of fly and female frog preferences within species would likely shed light on how opposing selection pressures impact signal evolution. An analysis of the relative linearities of the body size and call pitch correlation among frog species that are and are not attacked by flies may validate (or invalidate) the idea that small frogs might increase pitch beyond what their biomechanical constraints would explain in response to greater vulnerability to blood loss or infection load effects. Other acoustic signal traits that impact attraction to both target and non‐target receivers should also be tested: interspecific variation in complexity (via temporal, spectral and/or amplitude modulation), duty cycle (via note repetition rate and note or internode interval duration) and harmonics. Testing these additional variables may also reveal the elements of frog calls that serve as cues for the Australian *Sycorax* and *Uranotaenia*.

To our knowledge, this is the first study of signal preferences in any acoustic sexual signaller‐eavesdropper system in Oceania. By elucidating a strong spectral preference in an Australian frog‐biting midge, we have established this as a useful model for investigating the complex preference dynamics of intended and unintended signal receivers. We also shed light on potential impacts of these dynamics on evolution and population patterns of amphibians, the most threatened terrestrial vertebrates globally (Luedtke et al. 2023). Finally, further studies involving frog‐biting midges are poised to have methodological success, with knowledge of specific frequencies that can attract them in large numbers and the potential ecological factors behind these.

## Author Contributions

**Timothy P. Cutajar:** conceptualization (lead), data curation (lead), formal analysis (equal), investigation (lead), methodology (lead), project administration (lead), visualization (lead), writing – original draft (lead), writing – review and editing (equal). **Alistair G. B. Poore:** formal analysis (equal), investigation (equal), methodology (equal), supervision (lead), visualization (supporting), writing – review and editing (equal). **Jodi J. L. Rowley:** conceptualization (supporting), investigation (equal), methodology (equal), supervision (lead), visualization (supporting), writing – review and editing (equal).

## Conflicts of Interest

The authors declare no conflicts of interest.

## Supporting information


**Data S1:** ‘No choice experiment data_202302.csv’ (provided separately).

**Data S2:** Experimental code (provided separately).

## Data Availability

The data that support the findings of this study and its experimental R code are available in the Supporting Information of this article.

## Appendix A

To determine the range of frequency treatments used during phase one of our trapping experiment, we downloaded up to five call recordings from the FrogID dataset (Rowley and Callaghan 2020) for each of the nine frog species detected in the study area by Cutajar and Rowley (2020). Though Cutajar and Rowley (2020) determined 28 frog species to be known from within 10 km of the study sites but detected only nine, 17 of the 19 they did not detect are not associated with perennial wet sclerophyll and rainforest streams, and the few nearby records of the other two ( *Mixophyes fasciolatus* and *M. iteratus* ) have poor spatial precision (ALA 2024). Therefore, this nine‐species assemblage is representative of the study area.

Recordings were randomly chosen after filtering for recordings with the ‘quality call’ tag (signalling calls of sufficient quality for use in research), rejecting recordings with high levels of background noise. For species with fewer than five ‘quality call’ recordings in the dataset (only *Lechriodus fletcheri* ) we used all those available on 5 October 2021 (*n* = 3). Then from each recording we measured the dominant frequency of five individual consecutive calls, defined here as a vocalisation produced during a discrete expiration (Brown and Richards 2008). We used Raven Pro v.1.6.1 software (http://www.birds.cornell.edu/raven) with fast‐Fourier transform of 512 points and 50% overlap using Hanning windows after converting recordings with a sampling rate of 44.1 kHz from AAC to WAV format using the Mediahuman Audio Converter (https://www.nch.com.au). We then calculated the mean for each recording and used those values to calculate means for each species. The mean, minimum and maximum dominant frequency (kHz) for each of the nine frog species are visualized in Figure 1 and were: *Adelotus brevis* : 1.1 (0.5–1.8); *Crinia signifera* : 2.9 (2.4–3.2); *Lechriodus fletcheri* : 0.8 (0.6–1); *Litoria barringtonensis* : 2.2 (1.9–2.9); *L. chloris* : 1.8 (1.5–3); *L. daviesae* : 1.3 (1–1.6); *L. phyllochroa* : 2.7 (2.1–3); *L. wilcoxii* : 1.2 (1–1.3); and *Mixophyes australis* (referred to as *M. balbus* in Cutajar and Rowley (2020) prior to its split from that species following taxonomic treatment): 0.8 (0.6–1.5).
